# Leveraging Explainable Automated Machine Learning (AutoML) and Metabolomics for Robust Diagnosis and Pathophysiological Insights in Myalgic Encephalomyelitis/Chronic Fatigue Syndrome (ME/CFS)

**DOI:** 10.3390/diagnostics15212755

**Published:** 2025-10-30

**Authors:** Fatma Hilal Yagin, Cemil Colak, Fahaid Al-Hashem, Sarah A. Alzakari, Amel Ali Alhussan, Mohammadreza Aghaei

**Affiliations:** 1 Department of Biostatistics, Faculty of Medicine, Malatya Turgut Ozal University, 44210 Malatya, Türkiye; 2 Department of Computer Science, Lakehead University, Thunder Bay, ON P7B 5E1, Canada; 3 Department of Biostatistics, and Medical Informatics, Faculty of Medicine, Inonu University, 44280 Malatya, Türkiye; 4 Department of Physiology, College of Medicine, King Khalid University, Abha 61421, Saudi Arabia; 5 Department of Computer Sciences, College of Computer and Information Sciences, Princess Nourah bint Abdulrahman University, Riyadh 11671, Saudi Arabia; 6 Department of Ocean Operations and Civil Engineering, Norwegian University of Science and Technology (NTNU), 7034 Alesund, Norway

**Keywords:** Myalgic Encephalomyelitis/Chronic Fatigue Syndrome, metabolomics, Automated machine learning, explainable artificial intelligence, TPOT

## Abstract

**Background/Objectives**: Myalgic Encephalomyelitis/Chronic Fatigue Syndrome (ME/CFS) is a debilitating complex disease with an elusive etiology, lacking objective diagnostic biomarkers. This study leverages advanced Automated Machine Learning (AutoML) to analyze plasma metabolomic and lipidomic profiles for the purpose of ME/CFS detection. **Methods**: We utilized a publicly available dataset comprising 888 metabolic features from 106 ME/CFS patients and 91 matched controls. Three AutoML frameworks—TPOT, Auto-Sklearn, and H2O AutoML—were benchmarked under identical time constraints. Univariate ROC and PLS-DA analyses with cross-validation, permutation testing, and VIP-based feature selection were applied to standardized, log-transformed omics data to identify significant discriminatory metabolites/lipids and assess their intercorrelations. **Results**: TPOT significantly outperformed its counterparts, achieving an area under the curve (AUC) of 92.1%, accuracy of 87.3%, sensitivity of 85.8%, and specificity of 89.0%. The PLS-DA model revealed a moderate but statistically significant discrimination between ME/CFS and controls. Explainable artificial intelligence (XAI) via SHAP analysis of the optimal TPOT model identified key metabolites implicating dysregulated pathways in mitochondrial energy metabolism (succinic acid, pyruvic acid, leucine), chronic inflammation (prostaglandin D_2_, 11,12-EET), gut–brain axis communication (glycocholic acid), and cell membrane integrity (pc(35:2)a). **Conclusions**: Our results demonstrate that TPOT-derived models not only provide a highly accurate and robust diagnostic tool but also yield biologically interpretable insights into the pathophysiology of ME/CFS, highlighting its potential for clinical decision support and elucidating novel therapeutic targets.

## 1. Introduction

Myalgic Encephalomyelitis/Chronic Fatigue Syndrome (ME/CFS) is a serious, chronic, and multifactorial disease characterized by profound fatigue, post-exertional malaise (PEM), cognitive dysfunction, sleep disturbances, and autonomic symptoms (Institute of Medicine, 2015, https://www.cdc.gov/me-cfs/hcp/diagnosis/iom-2015-diagnostic-criteria-1.html, accessed on 18 September 2025). Its diagnosis remains challenging, relying primarily on clinical criteria after excluding other potential causes, due to the absence of definitive laboratory tests or biomarkers. This diagnostic ambiguity often leads to delayed diagnosis, patient frustration, and inadequate clinical management. The precise etiology of ME/CFS is unknown, but research points to a complex interaction of genetic, immunological, infectious, metabolic, and neurological factors [1].

In recent years, high-throughput omics technologies have emerged as powerful tools for uncovering the biological underpinnings of complex diseases [2,3,4]. Metabolomics, the comprehensive study of small-molecule metabolites, provides a direct functional readout of cellular activity and physiological state, offering a unique window into the metabolic disruptions associated with ME/CFS. Several metabolomic studies have suggested perturbations in energy metabolism, including impairments in glycolysis, the tricarboxylic acid (TCA) cycle, and lipid metabolism, pointing towards a state of mitochondrial dysfunction and energetic crisis [5,6,7]. However, analyzing high-dimensional metabolomic and lipidomic data presents significant challenges for traditional statistical methods [8,9]. The sheer number of features, coupled with complex, non-linear interactions, requires sophisticated machine learning (ML) approaches. The process of building an effective ML model—encompassing data preprocessing, algorithm selection, hyperparameter tuning, and validation—is highly specialized and time-consuming. Automated Machine Learning (AutoML) seeks to address this by automating the end-to-end process of applying machine learning, making it accessible to domain experts while often discovering novel and high-performing pipelines that may be overlooked by human experts [10]. While AutoML has shown promise in various biomedical domains, its application to ME/CFS metabolomics and lipidomics remains underexplored. A critical gap exists not only in identifying a high-performance model but also in interpreting its predictions to gain biological insights. Explainable artificial intelligence (XAI) techniques, such as SHAP (SHapley Additive Explanations), combined with comprehensive exploratory data analysis (EDA), are crucial for improving interpretability and transforming a “black box” model into a biologically meaningful analytical framework [11]. These analyses improve interpretability and provide clearer visualization of metabolomic patterns in ME/CFS. Therefore, this study had three primary objectives: (1) To conduct comprehensive EDA, including fold change, correlation heatmap, and Partial Least Squares Discriminant Analysis (PLS-DA) evaluations, to uncover key metabolic variations and enhance the interpretive depth of the dataset; (2) To benchmark the performance of three leading AutoML frameworks—TPOT, Auto-Sklearn, and H2O AutoML—in classifying ME/CFS based on plasma metabolomic and lipidomic data; and (3) To employ SHAP analysis on the optimal model to identify the most impactful metabolites and lipids and elucidate the dysregulated biological pathways they represent, thereby advancing both the diagnostic and pathophysiological understanding of ME/CFS. This integrated approach of competitive AutoML benchmarking coupled with model explainability for pathophysiological discovery in ME/CFS represents a promising contribution to the field.

## 2. Materials and Methods

### 2.1. Participant, Data, and Ethics Standard

The study utilized a publicly available metabolite and lipid dataset derived from plasma samples collected from 106 ME/CFS cases and 91 controls [12]. In the original study, all ME/CFS cases were diagnosed according to both the 1994 CDC Fukuda criteria and the Canadian Consensus Criteria, with diagnosis rendered by a clinician. All patients underwent standardized screening including medical history review, and physical examination. Stringent exclusion criteria were applied to eliminate comorbid conditions including chronic infections, rheumatic and inflammatory diseases, neurological disorders, psychiatric conditions, and use of immunomodulatory medications. The mean disease duration was 15.0 ± 9.8 years (range: 1.2–44.2 years). Blood samples were collected using BD Vacutainer™ Cell Preparation Tubes (Becton, Dickinson and Company, Franklin Lakes, NJ, USA) with EDTA anticoagulant following overnight fasting. Samples were immediately centrifuged, aliquoted, and stored at −80 °C until analyzed within two years of collection. Controls were frequency-matched to ME/CFS cases on gender, age, geographic/clinical region, race/ethnicity, and sampling date (±30 days) to minimize temporal variability and ensure comparability between groups. The current study was conducted in accordance with the principles of the Declaration of Helsinki and was approved by the Malatya Turgut Ozal University Health Sciences Scientific Research Ethics Committee (protocol code = 2025/252, 30 July 2025). The dataset comprised 888 metabolic analytes including metabolites, biogenic amines, complex lipids, and oxylipins, obtained through three mass spectrometry platforms: gas chromatography/time-of-flight mass spectrometry (GC-TOF MS), hydrophilic interaction liquid chromatography/quadrupole time-of-flight mass spectrometry (HILIC-QTOF MS), and liquid chromatography/quadrupole time-of-flight mass spectrometry (CSH-QTOF MS) [12].

### 2.2. Data Preprocessing

Prior to model training, panel data containing metabolomics and lipidomics related to ME/CFS patients underwent a comprehensive cleaning process. In particular, rows containing missing values related to omics levels were processed to reduce bias and ensure accuracy. In data analytics processes, it is important to address missing data when encountered and to make the dataset suitable for further analysis. In omics data, missing values can affect analysis results, so it is necessary to assign values to these data using an appropriate method. In this study, omics with a missing rate of over 30% were excluded and not included in further analyses. The miceforest [13] was used to estimate missing omics levels with a missing rate of less than 30%. Missing values were estimated using the Multiple Imputation with Chain Equations (MICE) [14] approach based on the LightGBM [15] model. This approach, which combines the power of MICE with LightGBM, provides computational speed, memory efficiency, and data type flexibility for missing value estimation. To handle missing values, the miceforest was employed, executing 5 imputation runs with 100 iterations each to generate a stable, consolidated imputed dataset. To ensure that all features contribute equally to model performance and to increase generalizability, we examined the different scaling conditions of omics levels. At this stage, we applied scaling to standardize omics values within a common range. This prevents larger-scale features from dominating the model and ensures that all features have a proportional effect. For feature scaling, the StandardScaler from the scikit-learn library was applied to standardize all omics values, transforming them to a distribution with a mean of zero and a standard deviation of one, ensuring no feature disproportionately influenced the model due to its original scale.

### 2.3. Machine Learning Pipeline

To ensure robust and generalizable performance estimates, the dataset was subjected to Repeated Random Sub-sampling Validation. Specifically, the split into 80% training and 20% testing sets was repeated 100 times, with performance metrics reported as the mean and standard deviation across all iterations.

Each AutoML framework was configured with a strict, identical time budget of 600 s for pipeline search:

TPOT: The TPOTClassifier was initialized with a population_size of 100 and a generation count of 10, leveraging its evolutionary algorithm to explore a wide range of preprocessing operators, feature selectors, and classification models.

Auto-Sklearn: The AutoSklearnClassifier was used with time_left_for_this_task = 600 s and per_run_time_limit = 60 s, allowing its Bayesian optimization to efficiently search its model library.

H2O AutoML: The H2OAutoML function was executed with max_runtime_secs = 600, utilizing its randomized search and stacked ensemble strategy.

**Figure 1:**
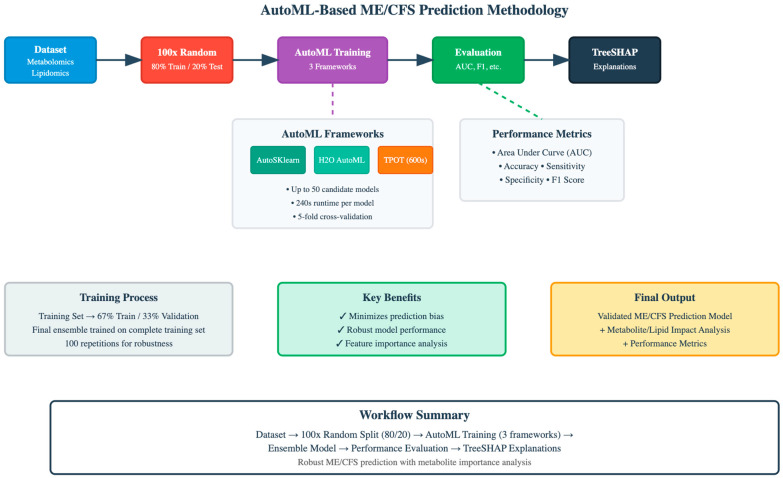
AutoML and XAI framework in ME/CFS explainable prediction.

The final pipeline identified by TPOT was refit on the entire 80% training partition before its final evaluation on the strictly held-out 20% test set [16,17,18]. Model performance was assessed using the area under the curve (AUC), accuracy, sensitivity, specificity, and F1 score. TPOT was allowed a maximum of 600 s to identify suitable machine learning pipelines. Within the training data, a default holdout strategy was applied, allocating 67% for training and 33% for validation. The ensemble modeling step considered up to 50 candidate models, each constrained to a runtime of 240 s. The final ensemble was then trained on the complete training set using 5-fold cross-validation. XAI represents a fundamental field of study focused on enhancing how we understand and interpret artificial intelligence systems. The primary goal is to illuminate how AI models reach their conclusions, fulfilling the essential requirement for trustworthiness and clarity in AI deployments throughout diverse industries. XAI employs a range of techniques designed to make machine learning algorithms more understandable, particularly for sophisticated model architectures. While simpler approaches like linear regression and decision trees offer natural interpretability through their straightforward structure, deep neural networks present significant challenges due to their multi-layered, complex computational processes. Research by Somani et al. [19] demonstrates that methods utilizing gradient information play a vital role in reconciling the tension between achieving strong prediction accuracy and maintaining model explainability. Therefore, this study employs TreeSHAP to enhance the interpretability of complex AutoML approaches. TreeSHAP is an efficient algorithm that computes SHAP values specifically optimized for tree-based ensemble models, providing fast and accurate feature importance explanations. This method enables us to understand how individual features contribute to predictions in AutoML frameworks, thereby making complex automated model selection and optimization processes more transparent and trustworthy. The AutoML and XAI framework used in the study is shown in Figure 1.

### 2.4. Statistical Analysis

To enhance analytical transparency, an EDA framework was integrated in addition to inferential modeling. This framework included fold change (FC) analysis, correlation heatmap visualization, univariate ROC analysis, and PLS-DA. FC analysis was performed to identify differentially expressed metabolites and lipids between ME/CFS patients and healthy controls. Statistical analysis included calculation of log_2_ fold changes (log_2_FC) and adjustment of *p*-values for multiple testing correction. Metabolites with adjusted *p*-values < 0.05 were considered statistically significant; positive log_2_FC values calculated upregulation, and negative log_2_FC values calculated downregulation. Results for compounds showing significant expression changes in FC analysis applied to 888 compounds are presented. To evaluate linear discriminatory patterns in the omics data, we applied univariate ROC curve analysis and PLS-DA model on standardized, logarithmically transformed omics concentrations. The optimal number of latent components was determined through 5-fold cross-validation, balancing model complexity with predictive performance. Log_10_ transformation followed by Z-score standardization was applied, and the PLS-DA model was implemented. We calculated the cumulative explained variance in class labels and VIP scores. Model significance was assessed through permutation testing (n = 200 iterations). Class labels were randomly shuffled, and the model was refitted to produce the null distribution of R^2^Y values. The empirical *p*-value was calculated as the proportion of permuted R^2^Y values that exceeded the R^2^Y value of the original model. A *p*-value less than 0.05 was considered statistically significant. Metabolites with a VIP score ≥ 1.0 were considered discriminatory features, indicating that their contribution to class discrimination exceeded the average across all variables. Finally, correlation heatmap analysis was performed to examine patterns of association between variables. All analyses in this study were performed in Python 3.10 software using Spyder 6.0.7, an interactive workspace for data processing.

## 3. Results

The demographic and physical characteristics of the ME/CFS group (n = 106) and the control group (n = 91) were comparable, and to minimize potential confounding, the results reflected matching of controls to cases on age, sex, and BMI. The proportion of women and men was similar between groups (ME/CFS: 75 women [70.8%, 31 men [29.2%]; Controls: 69 women [75.8%, 22 men [24.2%]; *p* = 0.42). Mean age did not differ significantly (ME/CFS: 47.8 ± 13.7 years; Controls: 47.0 ± 14.1 years; *p* = 0.78) nor did mean BMI (ME/CFS: 26.1 ± 5.2; Controls: 25.2 ± 4.7; *p* = 0.31). These findings confirm that the two groups were well balanced with respect to these key baseline variables (Table 1).

The results of FC analysis applied to metabolomic and lipidomic biomarker candidate compounds are presented in Table 2. According to the integrated lipidomic and metabolomic data and FC analysis results, 28 significantly altered lipid species and metabolites (adj *p* < 0.05) were identified between ME/CFS patients and healthy controls, of which 14 were up-regulated and 14 were down-regulated in ME/CFS patients. Among glycerophospholipids, phosphatidylcholines (pc’s) showed predominantly decreased levels in ME/CFS, while seven pc species, including pc(32:2) (log_2_FC = −0.235, *p* = 0.004) and pc(p-34:2) or pc(o-34:3) (log_2_FC = −0.255, *p* = 0.007), were significantly down-regulated in ME/CFS patients. However, two pc species were up-regulated. Phosphatidylethanolamines showed significant decreases in plasmalogen species pe(p-34:2) or pe(o-34:3) (log_2_FC = −0.219, *p* = 0.021) and pe(p-36:2) or pe(o-36:3) (log_2_FC = −0.195, *p* = 0.018). Sphingolipid metabolism showed complex changes with distinct changes in ceramide species including significant increase in sm(d36:0) (log_2_FC = 0.319, *p* = 0.007) and upregulation of cer(d34:1) and cer(d42:2)a and downregulation of cer(d42:2)b. Glycosphingolipids were consistently elevated, with GlcCer(d42:2) showing the strongest significance (log_2_FC = 0.247, *p* = 0.001). Cholesterol ester profiling revealed a decrease in the polyunsaturated species ce(18:2) and ce(18:3), while an increase in the omega-3 fatty acid esters ce(20:5) (log_2_FC = 0.528, *p* = 0.021) and ce(22:6) (log_2_FC = 0.267, *p* = 0.004). Lysophosphatidylcholines, containing polyunsaturated fatty acids, lpc(18:2) and lpc(18:3), were significantly decreased in ME/CFS patients. Analysis of metabolomics data revealed upregulation of the TCA cycle intermediates succinic acid (log_2_FC = 0.185, *p* = 0.014) and threonic acid (log_2_FC = 0.209, *p* = 0.035), downregulation of the branched-chain amino acid leucine (log_2_FC = −0.160, *p* = 0.023), and increased levels of aminomalonate (log_2_FC = 0.296, *p* = 0.013). These findings suggest coordinated changes in membrane phospholipid remodeling, sphingolipid metabolism, fatty acid esterification patterns supporting omega-3 involvement, and energy metabolism and amino acid catabolism pathways (Table 2). In addition, the correlation heatmap shows the pairwise relationships between all measured omics levels. Overall, most omics showed weak to moderate positive correlations (Supplementary File Figure S1).

In this study, we benchmarked TPOT against other automated machine learning approaches—namely Auto-sklearn [20], and H2O AutoML [21]—using an identical time budget to that of TPOT. Auto-sklearn leverages Bayesian optimization to fine-tune the data pipelines it generates and, upon completing the search, compiles an ensemble of trained pipelines [22,23]. H2O AutoML, developed on the H2O platform, employs randomized search strategies and incorporates tailored algorithm configurations with early stopping to improve efficiency. Its design prioritizes a balance between inference speed and predictive accuracy, producing models suitable for real-world deployment.

The results obtained showed that TPOT achieved significantly superior performance compared to other models in all performance metrics. TPOT achieved the highest accuracy rate of 87.3 ± 2.3%, while also demonstrating balanced and high performance in terms of sensitivity (85.8 ± 3.2%) and specificity (89.0 ± 2.6%). This indicates that the model is reliable in both accurately identifying ME/CFS patients (high sensitivity) and avoiding misclassifying healthy individuals as false positives (high specificity). Additionally, TPOT outperformed other algorithms in terms of F1 score (87.9 ± 2.9) and AUC value (92.1 ± 1.9), indicating that the classification success is consistent in terms of both accuracy and discriminative power. AutoSklearn showed moderate performance in ME/CFS detection (AUC 83.7 ± 3.1), while H2O AutoML had the lowest accuracy and AUC values (70.1 ± 5.1 and 75.6 ± 4.5, respectively). Our comparative analysis of AutoML methods showed that TPOT achieved superior performance over Auto-sklearn and H2O AutoML across all evaluation metrics. TPOT consistently delivered high accuracy and AUC values, whereas the results from Auto-sklearn and H2O AutoML were slightly lower. Between the latter two, Auto-sklearn generally surpassed H2O AutoML, which trailed in most measures. These findings suggest that although each approach is capable of producing effective models, TPOT demonstrates greater overall robustness. From a clinical perspective, TPOT’s high sensitivity value supports its ability to capture disease-related biological signals, while its high specificity offers the potential to reduce unnecessary testing and misdiagnosis risks. Therefore, TPOT is considered a strong candidate for clinical decision support systems in the early and accurate detection of ME/CFS (Table 3).

**Figure 2:**
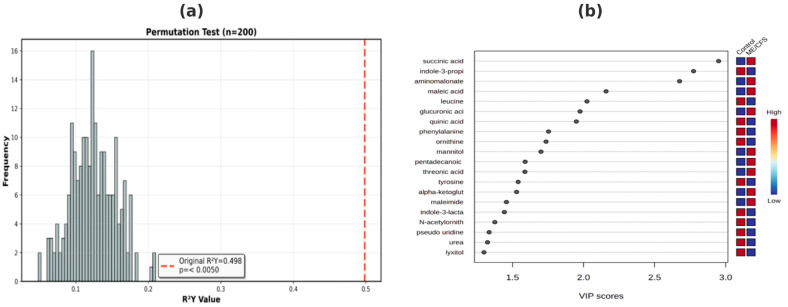
Permutation results and variable importance graph regarding the discrimination power of the PLS-DA model. (**a**): PLS-DA model permutation test results; (**b**): PLS-DA model VIP graph.

PLS-DA revealed a moderate but statistically significant class separation between ME/CFS patients and healthy controls (Figure 2a). The model explained 49.8% of the variance in disease status (R^2^Y = 0.498). Permutation testing (n = 200) confirmed model validity with high statistical significance (*p* < 0.005), demonstrating that the observed separation was not attributable to chance. This model reflects the known metabolic heterogeneity in ME/CFS, where patient subgroups can exhibit variable metabolic phenotypes. Ten component VIP scores showed ≥1.0 (Figure 2b), indicating strong discriminatory power. According to Figure 2b, leucine levels were decreased in ME/CFS, suggesting impaired branched-chain amino acid (BCAA) metabolism and possible mitochondrial dysfunction. Glutamine was increased in ME/CFS, consistent with immune activation and gut–brain axis dysregulation. Pyruvic acid was increased in ME/CFS, indicating impaired glycolysis-to-TCA cycle switching and possible metabolic inflexibility. Succinic acid was increased in ME/CFS, indicating TCA cycle defects and possible pseudohypoxia. These findings are consistent with established ME/CFS pathophysiology, including mitochondrial dysfunction, immune dysregulation, and altered energy metabolism. While PLS-DA identified biologically relevant metabolic patterns, the moderate R^2^ and partial overlap in the scorecards highlight the nonlinear, multifactorial nature of ME/CFS metabolic dysregulation. This motivated our application of advanced machine learning (TPOT AutoML), which achieved superior classification performance (87.3% accuracy) by capturing complex, high-dimensional omics interactions (Figure 2).

**Figure 3:**
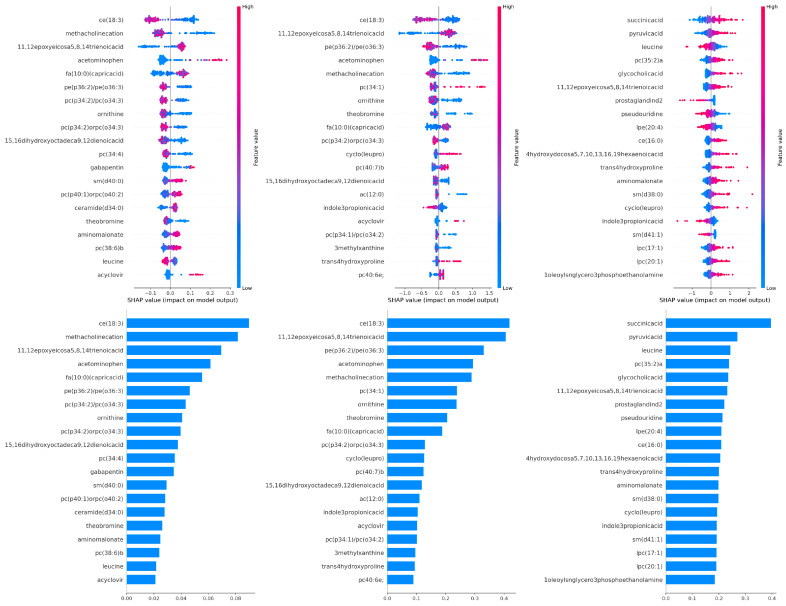
SHAP explanations for H2O AutoML, AutoSklearn, and TPOT models.

Figure 3 shows the SHAP analysis results for three different models (H2O AutoML, AutoSklearn, and TPOT, respectively). The horizontal scatter plots at the top of each panel visualize the metabolites that are most influential in the model’s prediction and the effect of changes in these metabolite levels on the model output (ME/CFS probability). The SHAP value indicates the direction and magnitude of the metabolite’s contribution to the model prediction; positive SHAP values to the right of zero indicate that an increase in that feature increases the probability of ME/CFS, while negative SHAP values to the left indicate that it decreases the probability. The color of the points represents the relative high (red) or low (blue) level of the metabolite. The concentration of red points in positive SHAP values indicates that an increase in the level of the relevant metabolite increases the probability of ME/CFS, while the presence of blue points on the positive side indicates that low levels increase this probability. The bar graphs at the bottom of each panel rank the average contribution size of metabolites to the model prediction based on mean(|SHAP value|); this allows the importance of metabolites to be compared independently of their contribution.

In our metabolomic and lipidomics analysis, the SHAP evaluation of our optimal model, TPOT, clearly identifies three fundamental biological axes that explain the pathophysiology of ME/CFS. The metabolites that stood out in the graph were primarily succinic acid, pyruvic acid, leucine, pc(35:2)a, glycocholic acid, 11,12-epoxyeicosa-5,8,14-trienoic acid, prostaglandin D_2_, and pseudouridine. Based on these findings, it was determined that increased levels of succinic acid, pyruvic acid, pc(35:2)a, glycocholic acid, and 11,12-epoxyeicosa-5,8,14-trienoic acid, along with decreased levels of leucine, prostaglandin D_2_, and pseudouridine, increase the likelihood of developing ME/CFS. The increase in succinic acid levels, indicating disruption in the TCA cycle, and the increase in pyruvic acid levels, reflecting metabolic overload in the glycolysis-TCA transition, support the notion of inefficient energy metabolism and the possibility of mitochondrial bottlenecks in ME/CFS. In contrast, decreased levels of leucine indicate increased catabolism in branched-chain amino acid (BCAA) metabolism and its association with neuromuscular fatigue. In the chronic inflammation axis, decreased levels of the pro-inflammatory lipid mediator prostaglandin D_2_ suggest that the inflammatory response may be suppressed or shifted to a different pathway; an increase in 11,12-Epoxyeicosa-5,8,14-trienoic acid levels suggests that potential anti-inflammatory or vascular protective mechanisms may be activated. The increase in pc(35:2)a, indicating impaired phospholipid dynamics, reflects adaptive or dysfunctional remodeling of cell membrane composition and membrane fluidity; while the increase in bile acid glycocholic acid points to impaired interactions between the gut microbiota–bile acid metabolism–brain axis. The decrease in pseudouridine levels suggests that RNA catabolism may be suppressed, potentially leading to a weakening of gut–brain axis communication (Figure 3).

In addition, univariate ROC analyses showed moderate discrimination power for the evaluated first important three compounds in TPOT SHAP result (Supplementary File Figure S2). Figure S2a produced an AUC of 0.611, indicating that succinic acid achieved limited but consistent discrimination. Figure S2b, pyruvic acid performed slightly better with an AUC of 0.618, indicating comparable but slightly stronger discrimination ability. Figure S2c, leucine produced an AUC of 0.607, supporting the general trend of moderate classification ability. Although none of the models reached a high AUC threshold (≥0.80), all three achieved AUC values above 0.60, reflecting statistically significant but modest predictive performance. Thus, individual omics showed limited discriminatory power, indicating that no single feature alone could robustly distinguish groups. However, after integrating multiple metabolites using the TPOT AutoML pipeline, model performance substantially improved (AUC = 92.1), highlighting the synergistic predictive value of combined features (Figure S2).

## 4. Discussion

The current study presents a comprehensive methodological framework that significantly advances the diagnostic and pathophysiological understanding of ME/CFS by integrating competitive AutoML benchmarking with XAI, in addition to exploratory data analysis to uncover biological interactions. The integration of EDA further enhanced the interpretability of our results, allowing clearer visualization of metabolite interactions and providing supportive evidence for the robustness of our methodological framework. While previous metabolomic and lipidomic studies in ME/CFS have primarily relied on conventional statistical comparisons or applied single, pre-specified machine learning models, our work is the first to systematically evaluate and benchmark multiple leading AutoML paradigms under strict, identical time constraints. This rigorous comparative approach not only identified TPOT’s evolutionary algorithm as the superior strategy for navigating the high-dimensional complexity of the ME/CFS metabolome but also underscores the critical importance of the search strategy itself in biomarker discovery. The principal novelty, however, extends beyond superior classification performance. We leverage the optimally performing TPOT model not as an impenetrable black box, but as a powerful discovery engine through subsequent SHAP analysis. This crucial step transforms a high-accuracy classifier into a biologically interpretable tool, enabling the data-driven identification and prioritization of dysregulated pathways—including mitochondrial energy metabolism, chronic inflammation, gut–brain axis communication, and cell membrane integrity. This dual-pronged methodology, which competitively seeks the most robust predictive pipeline and then extracts mechanistic insights from it, provides a replicable and powerful blueprint for deconstructing the complexity of ME/CFS and other enigmatic chronic diseases, ultimately bridging the gap between computational prediction and clinical etiological understanding.

This study sought to evaluate the efficacy of advanced AutoML frameworks in developing a robust diagnostic model for ME/CFS based on plasma metabolomic and lipidomic profiles. Our findings demonstrate that the TPOT significantly outperformed both Auto-Sklearn and H2O AutoML across all performance metrics, achieving an impressive AUC of 92.1%, accuracy of 87.3%, and a balanced sensitivity and specificity of 85.8% and 89.0%, respectively. The superior performance of TPOT, coupled with the biological plausibility of the features it prioritized, underscores the potential of evolutionary algorithm-based AutoML and XAI in identifying complex, multifactorial diseases like ME/CFS.

PLS-DA successfully identified metabolic abnormalities consistent with established ME/CFS pathophysiology and achieved statistical significance (*p* < 0.005) despite significant biological heterogeneity. The uncoupling metabolites appear to act as disease mechanisms in mitochondrial dysfunction and impaired energy metabolism. However, the moderate R^2^ and partial class overlap achieved in PLS-DA highlight a critical limitation: linear methods may not fully capture the complex, nonlinear metabolic derangements of ME/CFS. This disease likely involves complex omics interactions and patient subtypes that make linear decomposition challenging. FC analysis supported these findings by revealing 28 significantly altered metabolites and lipids (14 upregulated and 14 downregulated); these primarily included disruptions in phosphatidylcholine, sphingolipid, cholesterol ester, and TCA cycle-related metabolites, reflecting coordinated dysregulation in membrane remodeling, fatty acid oxidation, and mitochondrial energy metabolism. This result motivated our evolutionary AutoML application, which explores nonlinear models and feature engineering processes. TPOT’s superior performance demonstrates that advanced machine learning is essential for robust ME/CFS classification and effectively “learns” the multidimensional metabolic signatures that distinguish patients from controls.

The primary strength of this analysis lies in the application of multiple AutoML paradigms under strict, equivalent time constraints, providing a fair benchmark for their performance in a high-dimensional omics context. Beyond benchmarking, the key novelty lies in leveraging the optimally performing model not as a black box, but as a discovery tool via XAI, to generate a biologically interpretable and multi-faceted pathophysiological model for ME/CFS. TPOT’s evolutionary search strategy, which explores a wide range of preprocessing steps, feature selectors, and model architectures, proved to be exceptionally well-suited for navigating the complex interactions within the metabolomic data. In contrast, while Auto-Sklearn’s Bayesian optimization is efficient, it may have been constrained by its fixed set of preprocessors and models in this specific dataset. H2O AutoML’s randomized search, though fast and scalable, yielded the lowest performance, suggesting that a more guided and extensive search, as employed by TPOT, is necessary to uncover the subtle but significant patterns indicative of ME/CFS pathophysiology. This aligns with the core premise of AutoML: to automate the most challenging aspects of machine learning, ultimately finding non-intuitive pipelines that surpass human-designed models [10,21].

Beyond mere predictive accuracy, the application of SHAP analysis was pivotal for interpreting TPOT’s model, transforming it from a “black box” into a tool for biological discovery. The SHAP results delineated a coherent metabolomic signature, implicating several key interconnected biological pathways in ME/CFS. The elevation of succinic acid and pyruvic acid points directly to a profound dysregulation in energy metabolism. Increased succinate, an intermediate of the TCA cycle, often accumulates under hypoxic or inflammatory conditions and can itself act as an inflammatory signal. The concurrent rise in pyruvate, the end-product of glycolysis, suggests a bottleneck at the critical junction between glycolysis and the TCA cycle, potentially indicative of mitochondrial dysfunction or impaired pyruvate dehydrogenase complex activity. This is a well-replicated finding in ME/CFS literature, supporting the hypothesis of an acquired metabolic inflexibility and cellular energy deficit [6,7,24].

The observed reduction in leucine levels further corroborates the theme of metabolic disturbance. As a BCAA, leucine is crucial for protein synthesis and energy production in muscle tissue. Its depletion suggests increased catabolism, potentially to fuel alternative energy pathways under conditions of metabolic stress, and is strongly associated with the pervasive neuromuscular fatigue experienced by patients. Furthermore, the lipidomic profile revealed significant insights. The decrease in prostaglandin D_2_ (PGD_2_), typically a pro-inflammatory mediator, was unexpected but may indicate an exhaustion or compensatory shift in the inflammatory response rather than a simple absence of inflammation. Conversely, the increase in 11,12-epoxyeicosa-5,8,14-trienoic acid (11,12-EET), an epoxide derived from arachidonic acid with generally vasodilatory and anti-inflammatory properties, might represent an endogenous attempt to counter vascular dysfunction and inflammation. This complex, dysregulated lipid mediator profile paints a picture of a chronic, maladaptive immune response rather than acute inflammation [25,26].

The findings also strongly implicate the gut–brain axis in ME/CFS pathology. The increased level of the bile acid glycocholic acid suggests alterations in gut microbiota composition and function, as bile acids are metabolized by gut bacteria. Dysregulated bile acid metabolism can influence systemic inflammation, neuroendocrine signaling, and brain function via the farnesoid X receptor (FXR) and TGR5 receptors, providing a plausible mechanistic link between gut dysbiosis and the neurocognitive symptoms (“brain fog”) of ME/CFS. This is complemented by the decrease in pseudouridine, a modified nucleoside often linked to RNA turnover. Altered pseudouridine levels have been proposed as a marker of immune activation and cellular turnover, and its reduction could reflect broader disruptions in cellular metabolism and inter-organ communication [27,28]. Finally, the alteration in the phospholipid pc(35:2)a highlights membrane dysfunction. Phosphatidylcholines are fundamental components of cell membranes, and their composition determines membrane fluidity, signal transduction, and apoptosis. Changes in specific phospholipid species can indicate oxidative stress, inflammatory processes, and general cell membrane instability, which could affect neuronal and immune cell function throughout the body [29,30].

The clinical implications of these findings are substantial. The high specificity (89.0%) of the TPOT model is particularly crucial, as it minimizes the risk of false positives, thereby reducing the potential for unnecessary and invasive diagnostic procedures for healthy individuals. The high sensitivity (85.8%) ensures that the vast majority of true ME/CFS cases are identified, facilitating earlier intervention and support. The model’s ability to quantify the contribution of individual metabolites moves the field beyond simple biomarker discovery towards a functional, pathway-based understanding of the illness. This could not only aid in diagnosis but also in patient stratification (subtyping) and the identification of targeted therapeutic avenues, such as modulators of mitochondrial function, bile acid metabolism, or specific inflammatory pathways [31].

Despite these promising results, several limitations must be acknowledged. First, the sample size, though reasonable, remains modest for a high-dimensional omics study. External validation in a larger, independent cohort is essential to confirm the generalizability of the model and the identified metabolite signatures. Second, while MICE imputation is a robust method for handling missing data, the possibility of introducing bias cannot be entirely ruled out. Third, the cross-sectional nature of the data allows for the identification of associations but not causal relationships. An important limitation is that the dataset did not capture patients’ real-time clinical status at the time of blood sampling. ME/CFS patients commonly experience fluctuating symptom severity, including PEM episodes and ‘good’ vs. ‘bad’ days. Previous research has shown that metabolomic profiles can vary based on patient symptom state. The lack of real-time clinical status documentation represents a potential source of heterogeneity in our results. Future prospective studies should implement standardized sampling protocols aligned with patient “symptom states and incorporate longitudinal sampling to account for disease fluctuations. Longitudinal studies tracking metabolite levels before, during, and after symptom onset would be invaluable. Future work should focus on expanding the cohort, integrating multi-omics data (e.g., genomics, proteomics) to build a more comprehensive model, and exploring the potential of the identified metabolites as therapeutic targets.

## 5. Conclusions

In conclusion, this study demonstrates that the TPOT AutoML framework, empowered by XAI and EDA, is a powerful tool for distilling complex metabolomic data into a highly accurate and clinically interpretable diagnostic model for ME/CFS. The model’s performance and the biological coherence of its explanatory features provide strong support for the role of disrupted mitochondrial energy generation, altered lipid mediator metabolism, gut–brain axis dysregulation, and cell membrane instability in the pathophysiology of ME/CFS. This approach offers a promising pathway towards developing objective diagnostic tools and uncovering novel biological insights for this debilitating and often misunderstood disease.

## Figures and Tables

**Table 1:** Descriptive statistics on participants.

Characteristic	ME/CFS	Controls	*p*-Value
Gender: Female	75 (70.8%)	69 (75.8%)	0.42
Gender: Male	31 (29.2%)	22 (24.2%)	
Age (Mean ± SD)	47.8 ± 13.7	47.0 ± 14.1	0.78
BMI (Mean ± SD)	26.1 ± 5.2	25.2 ± 4.7	0.31

**Table 2:** Univariate statistical and fold change analysis results.

Metabolite/Lipid Name	log_2_FC	*p*-Value	Regulation
GlcCer (d42:2)	0.214	0.004	Up
pc(32:2)	−0.235	0.004	Down
pc(36:4)a	−0.173	0.006	Down
pc(40:6)b	0.207	0.004	Up
pc(p-34:1) or pc(o-34:2)a	−0.192	0.010	Down
pc(p-34:2) or pc(o-34:3)	−0.255	0.007	Down
pc(p-36:2) or pc(o-36:3)	−0.215	0.007	Down
pc(p-38:3) or pc(o-38:4)	−0.110	0.033	Down
pc(p-40:4) or pc(o-40:5)	0.114	0.038	Up
pe(p-34:2) or pe(o-34:3)	−0.219	0.021	Down
pe(p-36:2) or pe(o-36:3)	−0.195	0.018	Down
sm (d36:0)	0.319	0.007	Up
ce(18:2)	−0.080	0.031	Down
ce(18:3)	−0.199	0.029	Down
ce(20:5)	0.528	0.021	Up
ce(22:6)	0.267	0.004	Up
cer(d34:1)	0.098	0.024	Up
cer(d42:2)a	0.142	0.017	Up
cer(d42:2)b	−0.143	0.029	Down
dg(36:2)	0.222	0.028	Up
GlcCer(d42:2)	0.247	0.001	Up
Lactosylceramide (d18:1/24:1(15Z))	0.171	0.019	Up
lpc(18:2)	−0.218	0.027	Down
lpc(18:3)	−0.260	0.025	Down
threonic acid	0.209	0.035	Up
succinic acid	0.185	0.014	Up
leucine	−0.160	0.023	Down
aminomalonate	0.296	0.013	Up

**Table 3:** Performance metrics related to AutoML model results in ME/CFS detection.

Metrics/Algorithms	TPOT	AutoSklearn	H2O AutoML
Accuracy	87.3 ± 2.3%	78.7 ± 3.7%	70.1 ± 5.1%
Sensitivity	85.8 ± 3.2%	75.5 ± 4.5%	67.0 ± 6.0%
Specificity	89.0 ± 2.6%	82.4 ± 4.0%	73.6 ± 4.9%
F1 score	87.9 ± 2.9%	79.2 ± 4.1%	70.6 ± 5.4%
AUC	92.1 ± 1.9%	83.7 ± 3.1%	75.6 ± 4.5%

## Data Availability

The raw data supporting the conclusions of this article will be made available by the authors on request.

## Supplementary Materials

The following supporting information can be downloaded at: https://www.mdpi.com/article/10.3390/diagnostics15212755/s1, Figure S1: Correlation heatmap analysis results; Figure S2: Univariate ROC curves of the first three biomarker candidate compounds in the TPOT SHAP plot.
