# Supplementary material for: Leveraging Explainable Automated Machine Learning (AutoML) and Metabolomics for Robust Diagnosis and Pathophysiological Insights in Myalgic Encephalomyelitis/Chronic Fatigue Syndrome (ME/CFS)
Source: Diagnostics (Basel). 2025 Oct 30;15(21):2755. doi: 10.3390/diagnostics15212755 (PMC12609673; doi:10.3390/diagnostics15212755)
Supplement: Supplementary file 1 [file diagnostics-15-02755-s001.zip › diagnostics-3918268-supplementary.pdf]

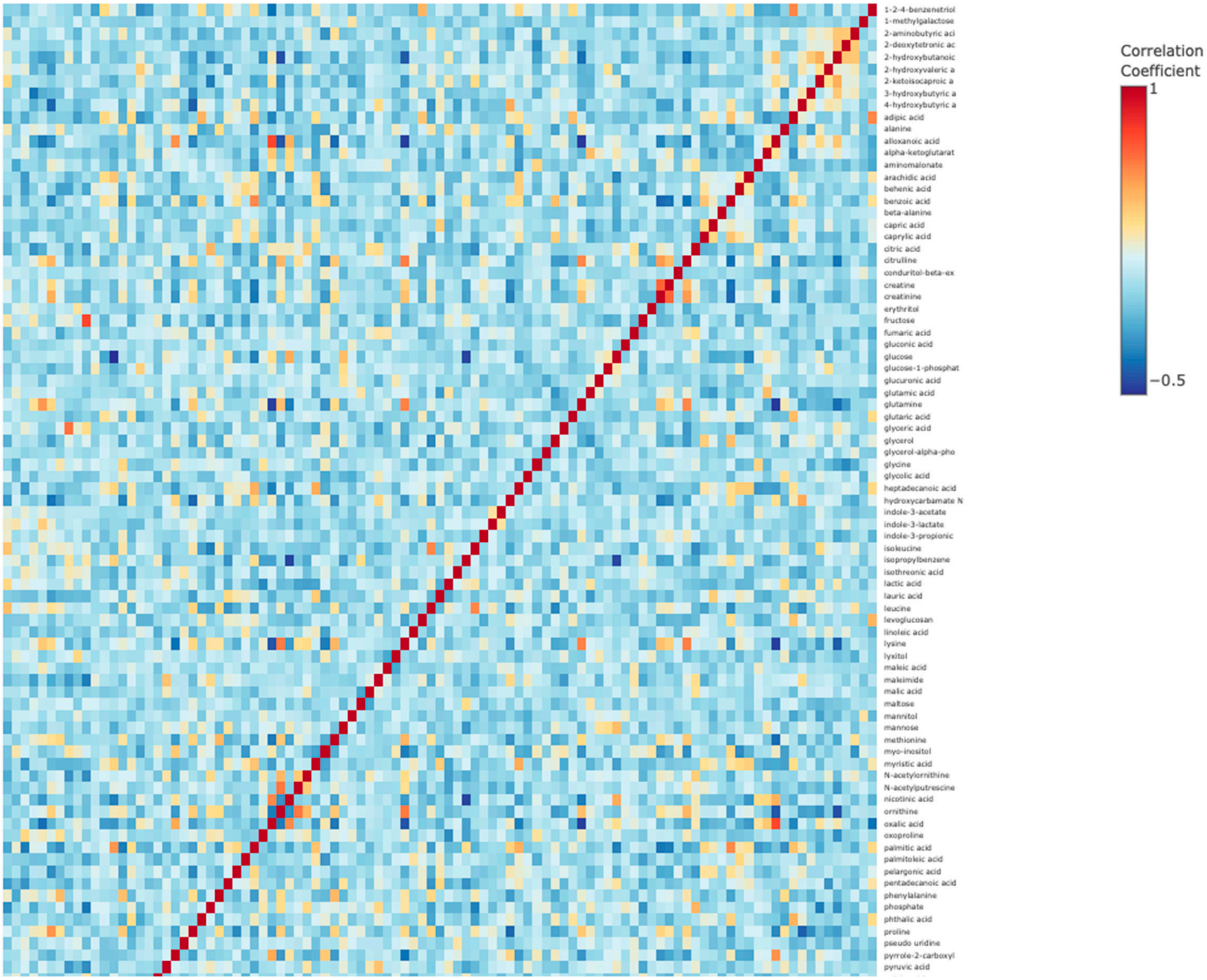

Figure S1. Correlation heatmap analysis results**.**

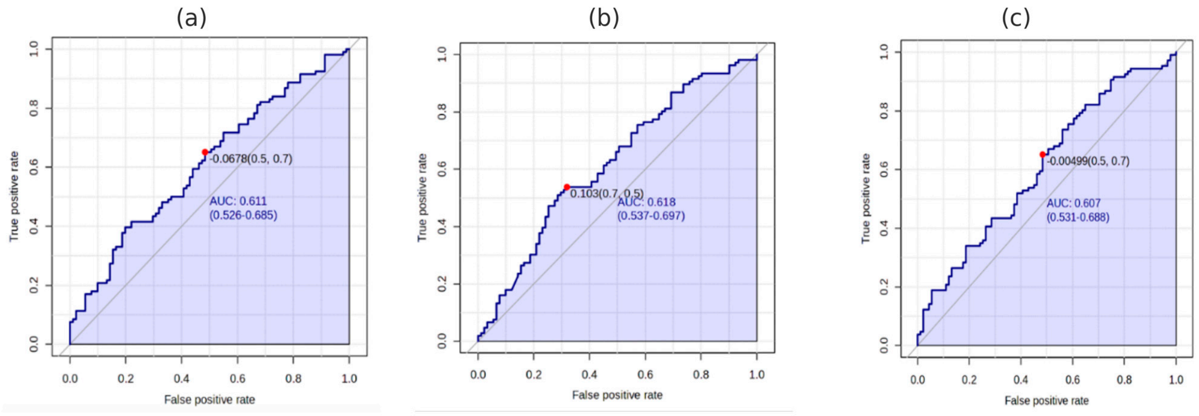

Figure S2. Univariate ROC curves of the first three biomarker candidate compounds in the TPOT SHAP plot.
